# Bioactive Compounds, Antioxidant Properties, and Antimicrobial Profiling of a Range of West Algerian Honeys: In Vitro Comparative Screening Prior to Therapeutic Purpose

**DOI:** 10.3390/foods13244120

**Published:** 2024-12-20

**Authors:** Dalila Bereksi-Reguig, Hocine Allali, Nadjat Taib, Nadia Aissaoui, Marzena Wlodarczyk-Stasiak, Radoslaw Kowalski

**Affiliations:** 1 Department of Chemistry, Faculty of Sciences, Aboubekr Belkaïd University, P.O. Box 119, Tlemcen 13000, Algeria; dalilabereksi13@gmail.com; 2 Laboratory of Biotoxicology, Pharmacognosy and Biological Valorization of Plants, Department of Biology, Faculty of Sciences, Doctor Tahar Moulay University, P.O. Box 138 cité EN-NASR, Saïda 20000, Algeria; nadjat.taib.dr@gmail.com; 3 Laboratory for the Sustainable Management of Natural Resources in Arid and Semi-Arid Areas, University Center Salhi Ahmed Naâma, Bp 66, Naâma 45000, Algeria; aissaoui.nadia@cuniv-naama.dz; 4 Department of Analysis and Food Quality Assessment, University of Life Sciences in Lublin, 8 Skromna Str., 20-704 Lublin, Poland; radoslaw.kowalski@up.lublin.pl

**Keywords:** honey, bioactive compounds, antioxidant activity, antimicrobial profiling

## Abstract

Honey is a complex natural nutrient with well-established therapeutic properties recognized in traditional medicine. The purpose of the current work was to compare, in vitro, the bioactive compounds, antioxidants, and antimicrobial properties of 37 honey samples collected from the western region of Algeria and to identify the best sample for potential therapeutic purposes. Estimation of bioactive compounds was carried out by determining the total phenolic and flavonoid contents. Large variability among the samples was observed regarding the total phenolic content (from 24.17 ± 1.38 to 122.15 ± 3.55 mg GAE/100 g honey) and total flavonoid content (from 0.07 ± 0.01 to 33.49 ± 4.90 mg QE/100 g honey). Additionally, antioxidant activity, evaluated by four spectrophotometric assays, displayed fluctuating results among the samples. High positive correlations were observed between β-carotene and DPPH (0.766) and between β-carotene and ABTS (0.600), while inverse correlations were observed between bioactive compounds and antioxidant activity, except for the FRAP method. The antimicrobial activity, determined by well-diffusion assays, exhibited a dose-dependent antibacterial effect, with significant inhibition toward methicillin-resistant *Staphylococcus aureus* and *Pseudomonas aeruginosa* reference strains. However, no activity was observed against *Candida albicans* strains. The MIC and MBC values were identical in most samples (range: 60 to 80% *w*/*v*) and predominantly exhibited bactericidal effects. The content of bioactive compounds played a significant role in the antibacterial properties. To summarize, the best honey sample for potential therapeutic purposes corresponded to mild white mustard (S6) and might be used as an alternative in therapeutic applications.

## 1. Introduction

Natural honey (NH) is a complex chemical nutrient primarily composed of saccharides (about 74–78%) and water (approximately 16–18%) [1]. NH also contains miscellaneous constituents, which are extremely complex and relatively minor, including complex saccharides, minerals, antioxidants, phenolic compounds, vitamins, proteins, enzymes, and some unidentified molecules [2]. These remaining compounds, which encompass the aforementioned constituents, determine the variability and biological activities of honey [3].

The therapeutic properties of NH are well known and it is introduced in medical settings to prevent and limit bacterial infections [4], cure wounds and burns [5], alleviate inflammation, and exhibit antioxidant effects, in addition to its food value [3]. Recent works investigating the therapeutic properties of NH focus on antioxidant and antimicrobial potencies [2,3]. Generally, these activities are related and influence each other, and their combination results in the remarkable health properties of honey [6]. The antioxidant activity is attributed to the presence of flavonoids, phenolic acids, enzymes (peroxidase and catalase), trace elements, and vitamins (C and E), some of which also contribute to its antimicrobial activity.

Flavonoids and phenolic acids are usually found in plants and classified as secondary metabolites. They are derived from three pathways: shikimate, pentose phosphate, and phenylpropanoid [3]. Furthermore, their bactericidal, antiallergenic, anti-inflammatory, anticancer, and anticoagulant activities must be emphasized, in addition to their antioxidant effect [7]. In addition to the antioxidant activity, the antimicrobial effect of NH arises from several critical determinants, including low water activity, high sugar content, hydrogen peroxide, polyphenol compounds, bee-derived proteins, bee defensin-1, and methylglyoxal [8]. The antimicrobial activity of NH has been widely investigated in vitro against a broad spectrum of microorganisms, including Gram-positive and Gram-negative bacteria, yeasts, and fungi [2,9,10,11]. Interestingly, no microorganisms have developed resistance mechanisms against the various types of honey studied, which include samples from diverse botanical and geographical origins [8,12].

The antioxidant and antimicrobial activities vary significantly and depend on the above key factors and/or the presence of each constituent. Numerous factors are directly related to the floral source, climate conditions, geographical location, and bee health [8], as well as storage conditions, processing, and harvest time [13]. Several studies have described the antioxidant and antimicrobial effects of NH [14,15,16]. Unfortunately, none have investigated these potencies simultaneously for Algerian honeys, and the data are very limited and focus mainly on the pollen profile and physicochemical quality [17,18,19,20] or botanical origin [21]. Beekeeping is intensively practiced in the northern region of Algeria, where the biodiversity of flora is significant, providing abundant resources for honey production. The melliferous area spans approximately 797,122 hectares and includes an estimated 3150 plant species [20]. Moreover, the climate is sufficiently favorable, characterized by hot summers with a dry period, while winter is mild and precipitation is estimated at 400–1000 mm [22]. In Algeria, the major honey sources come from *Citrus* spp., *Eucalyptus* spp., *Rosmarinus officinalis* L., *Helianthus annuus* L., and wild mustard [20]. Nevertheless, unusual and lesser-known honey types are produced, such as *Ziziphus* and *Euphorbia* honeys [23] and *Erica* spp. honey [24]. Based on these facts, the aims of this current work are fourfold: (i) to assess the content of bioactive compounds in 37 Algerian honeys collected from various parts of the western region, (ii) to evaluate their antioxidant activity, (iii) to examine their antimicrobial capacity against reference strains, and (iv) to establish the relation between the biological properties of NH and the content of bioactive compounds. The overall aim was to identify the Algerian honey with the most outstanding bioactive properties.

## 2. Materials and Methods

### 2.1. Reagents and Standards

All the chemicals and reagents were of analytical grade purity. 2,2-Diphenyl-1-picrylhydrazyl (DPPH), 2,2′-Azino-bis (3-ethylbenzothiazoline-6-sulfonic acid) (ABTS), β-carotene, and Folin–Ciocalteu reagent were supplied by Sigma-Aldrich^®^ (Darmstadt, Germany). Gallic acid, L-ascorbic acid, quercetin, butylated hydroxytoluene (BHT), and Trolox^®^ (6-hydroxy-2,5,7,8-tetramethylchroman-2-carboxylic acid) were purchased from Sigma-Aldrich^®^ (Darmstadt, Germany). For antimicrobial activity, Mueller-Hinton Agar (MHA) and Mueller-Hinton Broth (MHB) were obtained from Conda Pronadisa (Madrid, Spain), and Sabouraud Dextrose Agar (SDA) was supplied by Liofilchem^®^, Italy. Buffers and stock solutions were made with high-purity de-ionized Milli-Q water.

### 2.2. Honey Samples and Physico-Chemical Characterization

**Figure 1:**
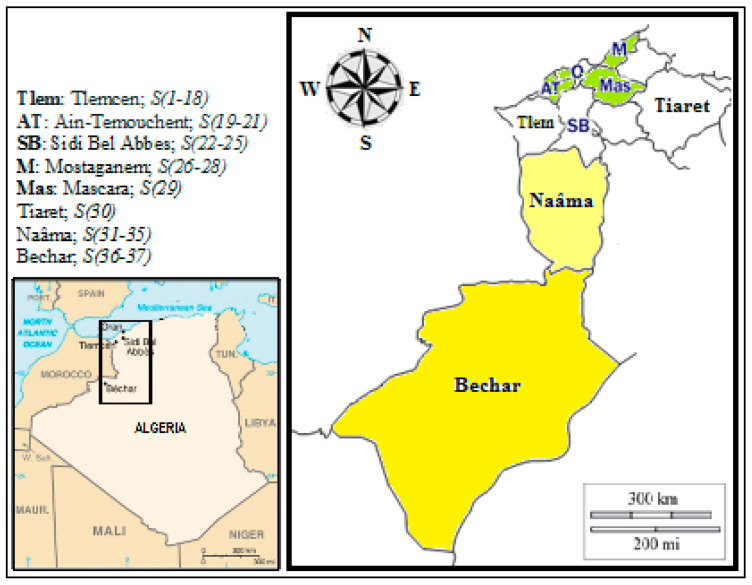
Map of the western region of Algeria showing the distribution of honey samples studied.

Thirty-seven NH samples of varying floral origin, identified as S(1–37), were obtained from experienced beekeepers in various geographical regions (Tlemcen, Ain-Temouchent, Sidi Bel Abbes, Mostaganem, Mascara, Tiaret, Naâma, and Bechar) located in the west of Algeria (Figure 1). The type and region (GPS coordinates, climate) of the honey samples, as well as the scientific and common names of the plants that form the basic flora of the honey samples, are shown in Table S1 as Supplementary Information. All samples were acquired between March 2017 and August 2018. Upon receipt, honeys were stored in darkness at ambient temperature until the start of the experiment. Electrical conductivity (EC) and pH were measured based on the methods described in the International Honey Commission (IHC) report [25,26].

### 2.3. Preparation of Honey Solutions

For antimicrobial assessments, two dilutions were prepared: 5 g of each honey was dissolved in either 10 mL or 6.25 mL of sterile distilled water to obtain weight-per-volume (*w*/*v*) concentrations of 50% and 80%, respectively.

### 2.4. Quantification of Bioactive Compound

#### 2.4.1. Determination of Total Phenolic Content (TPC)

The quantification of the TPC was carried out using the Folin–Ciocalteu method in an alkaline environment [27]. To 500 μL of Folin–Ciocalteu reagent, diluted tenfold with distilled water, 0.1 mL of honey solution (0.2 g/mL) was added, along with 400 μL of a 7% Na_2_CO_3_ solution. Following incubation for 40 min at room temperature, the absorbance was measured at a wavelength of 760 nm against a methanol blank using a UV-Visible spectrophotometer (OPTIZEN^TM^ POP, K Lab Co., Ltd., Daejeon, Republic of Korea). Methanolic solutions of gallic acid at various concentrations (0–1000 mg/L) were utilized as a standard to establish the calibration curve (y = 1.0815x − 14.445; R^2^ = 0.99). The results were expressed in milligrams of gallic acid equivalents (GAE) per 100 g of honey (mg GAE/100 g of honey).

#### 2.4.2. Determination of Total Flavonoids Content (TFC)

The TFC was estimated using the colorimetric aluminum chloride method [28]. One milliliter of honey solution in methanol (1 mg/mL) was mixed with 1 mL of a 2% Al_2_O_3_ solution in methanol. Following incubation for 40 min at room temperature, the absorbance of the yellow color produced by the reaction was measured at a wavelength of 430 nm using a UV-Visible spectrophotometer (OPTIZEN^TM^ POP, K Lab Co., Ltd., Daejeon, Republic of Korea). The flavonoid concentrations were determined from the calibration curve (y = 0.010x + 0.109; R^2^ = 0.96) constructed with quercetin as a standard at different concentrations (20, 40, 60, 80, and 100 mg/L). The results were expressed in milligrams of quercetin equivalents (QE) per 100 g of honey (mg QE/100 g of honey).

### 2.5. Determination of Antioxidant Activity

#### 2.5.1. Diphenyl-2-Picrylhydrazyl (DPPH) Radical Scavenging Activity

The radical scavenging potential of NH samples was evaluated using the DPPH assay as described by Sanchez-Moreno et al. [29]. The concentration of honey solutions ranged between 10 and 50 mg/mL. One milliliter of each honey solution was mixed with 1 mL of a methanolic solution of DPPH (0.12 mg/mL) and kept in darkness at room temperature under vigorous stirring for 60 min. The absorbance of the residual DPPH was then measured at a wavelength of 517 nm using a UV-Visible spectrophotometer (OPTIZEN^TM^ POP, K Lab Co., Ltd., Daejeon, Republic of Korea). Pure L-ascorbic acid at concentrations of 0.002–0.01 mg/mL was used as a positive control. The radical scavenging potential was estimated as a percentage of DPPH inhibition using the following Equation (1):

(1) $$\%DPPH\;inhibited=\frac{{Abs}_{control}\;-{Abs}_{sample}}{{Abs}_{control}}\;\times \;100$$

where

Abs_control_ is the absorbance of the control;
Abs_sample_ is the absorbance of the honey sample solution.

The IC_50_ values were calculated graphically (y = 3463x + 2.370; R^2^ = 0.98) by plotting the percentage of DPPH inhibition against the honey concentrations.

#### 2.5.2. Ferric Reducing Antioxidant Power (FRAP)

The FRAP of the honey samples was quantified using the method outlined by Karagözler et al. [30]. One milliliter of diluted honey in distilled water (10 mg/mL) was mixed with 2.5 mL of phosphate buffer (0.2 M, pH 6.6) and 2.5 mL of a 1% aqueous solution of potassium ferricyanide [K_3_Fe(CN)_6_]. The reaction mixture was incubated at 50 °C for 20 min. After cooling to room temperature, 2.5 mL of a 10% trichloroacetic acid solution was added, and the mixture was centrifuged at 3000 rpm for 10 min. Subsequently, a mixture of 2.5 mL of the supernatant, 2.5 mL of distilled water, and 0.5 mL of a 0.1% FeCl_3_ solution was prepared. The absorbance was read at a wavelength of 700 nm using a UV-Visible spectrophotometer (OPTIZEN^TM^ POP, K Lab Co., Ltd., Daejeon, Republic of Korea) after incubation for 10 min against distilled water as a blank. L-ascorbic acid was used for the calibration curve (y = 11.56x − 0.075; R^2^ = 0.98), and the results were expressed in milligrams of ascorbic acid equivalents per 100 g of honey (mg AAE/100 g).

#### 2.5.3. The Antiradical Activity of ABTS

The ABTS radical inhibition of the honey samples was evaluated using the method outlined by Bueno-Costa et al. [31]. A 7.0 mM ABTS aqueous solution was mixed with a 2.45 mM potassium persulfate solution in equal volumes. The resulting solution was incubated at room temperature in darkness for 16 h and then diluted with distilled water until a final absorbance of 0.70 ± 0.02 at a wavelength of 734 nm was achieved, as measured using a UV-Visible spectrophotometer (OPTIZEN^TM^ POP, K Lab Co., Ltd., Daejeon, Republic of Korea). Eighty microliters of diluted honey sample was mixed with 1 mL of the ABTS^+^. solution, and the absorbance was measured at 734 nm. The percentage inhibition of the ABTS⁺ radical cation was calculated using the following Equation (2):

(2) $$\%ABTS\;inhibited=\frac{{Abs}_{0}\;-\;{Abs}_{1}}{{Abs}_{0}}\;\times \;100$$

where

Abs_0_ is the absorbance of the control;
Abs_1_ is the absorbance of the test sample.

Standard solutions of Trolox (0.002–0.01 mg/mL) were used as the positive control. The IC₅₀ value was determined from the calibration curve (y = 2809x − 1.244; R^2^ = 0.99) by plotting the concentrations of honey samples against their corresponding inhibition effects (%ABTS inhibited).

#### 2.5.4. β-Carotene-Linoleic Acid Emulsion Method

The method for inhibiting β-carotene bleaching was performed as described in the literature [32]. Two milligrams of β-carotene was dissolved in 10 mL of chloroform, and 2 mL of this solution was pipetted into a small flask containing 20 mg of linoleic acid and 200 mg of Tween 40 emulsifier. The chloroform was then evaporated under vacuum at 40 °C, and 50 mL of hydrogen peroxide was added and stirred vigorously to form a fresh emulsion. One milliliter of the emulsion was transferred into test tubes containing 100 µL of honey samples diluted to different concentrations (10, 20, 30, 40, and 50 mg/mL). The absorbance of the mixtures was measured at a wavelength of 470 nm using a UV-Visible spectrophotometer (OPTIZEN^TM^ POP, K Lab Co., Ltd., Daejeon, Republic of Korea) immediately (*t* = 0 min) and after 120 min of incubation in a water bath at 50 °C against a blank (emulsion without β-carotene). The antioxidant activity of the honey samples was compared to butylated hydroxytoluene (BHT), used as a positive control. The β-carotene bleaching rate was calculated using the following Formula (3):

(3) $$Rate\;of\;\beta \;-\;carotene\;bleaching=ln\;\left(\frac{A_{0}}{A_{t}}\right)\;\times \;1/t$$

where

A_0_ is the initial absorbance of the emulsion at time 0;
A_t_ is the absorbance at time t;
t is the time in minutes.

The mean percentage inhibition of β-carotene bleaching was estimated using Equation (4):

(4) $$\left[\left(R_{control}/R_{sample}\right)/R_{control}\right]\;\times \;100$$

where

R_control_ is the bleaching rate of the β-carotene emulsion without an antioxidant;
R_sample_ is the bleaching rate of the β-carotene emulsion with the honey sample.

The IC₅₀ value was calculated using a standard curve (y = 11.64x + 0.086; R^2^ = 0.97) generated by plotting honey concentrations against the mean percentage inhibition of β-carotene bleaching.

### 2.6. Determination of Antimicrobial Activity

#### 2.6.1. Microbial Strains and Cultures Conditions

Six reference strains were utilized in this study, graciously provided by the Algerian Pasteur Institute. These strains included methicillin-susceptible *Staphylococcus aureus* (MSSA) ATCC 25923, methicillin-resistant *Staphylococcus aureus* (MRSA) ATCC 43300, *Pseudomonas aeruginosa* ATCC 27853, *Candida albicans* ATCC 10231, *Candida albicans* ATCC 26709, and *Candida albicans* IPP444. Routine cultures were performed aerobically for 24 h on Muller–Hinton Agar (MHA) for bacterial strains and on Sabouraud Dextrose Agar (SDA) for *Candida* species. Standardized inocula were prepared in 0.85% saline solution, with the densities adjusted to 10⁸ Colony Forming Units (CFU)/mL.

#### 2.6.2. Well-Diffusion Assay

To evaluate the antimicrobial activity of each NH, the well-diffusion assay was conducted as described by Ghramh et al. [33], with some modifications. Sterilized Muller–Hinton Agar (MHA) or Sabouraud Dextrose Agar (SDA) plates were swabbed with standardized bacterial or yeast inocula. Wells with a diameter of 5 mm were created using the sterile end of a Pasteur pipette and filled with 100 µL of each diluted honey sample or distilled water (negative control). The plates were then incubated at 37 °C for 24 h. Following incubation, the diameter of the inhibition zones (DIZ) around each well was measured in millimeters (mm). The NH samples were classified as follows: high activity (DIZ > 15 mm), moderate activity (DIZ: 12–15 mm), low activity (DIZ: 9–12 mm), very low activity (DIZ: 5.5–9 mm), and inactive (DIZ < 5.5 mm) [34]. Additionally, Gentamicin (10 µg) and Amphotericin B (10 UI) were tested against the microbial strains following the protocols outlined by the Clinical and Laboratory Standards Institute (CLSI) [35].

#### 2.6.3. Minimum Inhibitory Concentrations (MICs) and Minimum Bactericidal Concentrations (MBCs) Determination

Based on the results of the well-diffusion assay, the MICs were determined in sterile test tubes following the method described by Ghramh et al. [33], with some modifications. Seven different concentrations of NHs [5%, 10%, 20%, 40%, 60%, 80%, and 100% (*v*/*v*)] were prepared in 2 mL of Muller–Hinton Broth (MHB) as the final test volume. Each honey concentration was then inoculated with 20 µL of microbial suspension containing 10⁸ CFU/mL. After 24 h of incubation at 37 °C, the MIC was identified as the lowest concentration of honey at which no visible microbial growth was observed in the test tube. The MBCs were evaluated using the tubes from the MIC test that showed no visible growth. A 10 µL aliquot from each of these tubes was plated onto MHA and incubated aerobically at 37 °C for 24 h. The lowest concentration of honey that showed no growth on the MHA plates was recorded as the MBC.

### 2.7. Statistical Analysis

Statistical analyses were performed using SPSS (version 22.0). All tests were conducted in triplicate, and descriptive statistical analyses (means ± SD) were reported. Significant differences among the samples were determined using one-way ANOVA. The Duncan test was applied to compare the means at a significance level of *p* = 0.05. To group NH samples with relatively similar properties, cluster analysis methods were employed. Additionally, Pearson correlation analysis was conducted to evaluate the relationships between antioxidant methods and physicochemical parameters.

## 3. Results

### 3.1. Characterization of Honey Samples

The physicochemical parameters of the 37 NH samples are summarized in Table 1. In all samples, the pH and electrical conductivity values complied with the Codex Alimentarius and the European Community regulations. The pH was acidic, with values ranging between 3.38 ± 0.06 and 5.62 ± 0.04. The electrical conductivity values ranged from 0.165 ± 0.017 to 1.196 ± 0.021 mS/cm. Practically all the studied NHs were nectar-based (EC ≤ 0.8 mS/cm), except for three samples (S13, S19, and S20) which had a honeydew origin.

### 3.2. Quantification of Bioactive Compounds

The content of bioactive compounds in NHs varied significantly, depending on the botanical source and geographical region, as demonstrated in Table 1. The TPC varied across the NH types, with the highest content found in carob tree honey collected from Beni Ghazli (Tlemcen, Algeria) (122.15 ± 3.55 mg GAE/100 g, S12). In contrast, the lowest content was found in lavender honey (24.17 ± 1.38 mg GAE/100 g, S1). Regarding the TFC, the highest values were recorded in carob tree and multifloral honeys (33.49 ± 4.90 mg QE/100 g, S12; 26.49 ± 3.04 mg QE/100 g, S4), from Beni Ghazli (Tlem) and Sidi Djillali (Tlem), respectively. Conversely, the lowest amount was observed in lavender honey originating from Sidi Djillali (Tlem) (0.07 ± 0.01 mg QE/100 g, S1).

### 3.3. Assessment of Antioxidant Properties

Given the complex and diverse composition of NHs, which contain a wide array of bioactive compounds such as phenolic acids, flavonoids, enzymes, and trace elements, a single antioxidant assay may not adequately capture their full antioxidant potential. Each method provides insights into specific aspects of antioxidant activity: DPPH and ABTS assays evaluate the capacity to scavenge free radicals, the β-carotene-linoleic acid assay reflects the inhibition of lipid peroxidation, and the FRAP assay measures the ability to reduce ferric ions. The combined use of these complementary methods offers a more comprehensive and reliable assessment of antioxidant capacity, ensuring that both radical scavenging and redox properties are thoroughly evaluated. This multidimensional approach is essential for a robust characterization of honey’s antioxidant properties.

DPPH is a stable nitrogen-containing radical widely used to evaluate the free radical scavenging and antioxidant capacities of various compounds. Strong DPPH scavenging activity reflects significant antioxidant effectiveness in the sample. Among the NH samples, the EC_50_ of euphorbia honey from Ras El Ma (SB) (98.58 ± 6.29 mg/mL, S22) was the highest, while the lowest value was observed in multifloral honey from Oued es Safsâf (Tlem) (17.73 ± 1.53 mg/mL, S13) (Table 1).

The FRAP results revealed differences in antioxidant activity among the NH samples, indicating that the honeys exhibit varying levels of antioxidant capacity. Eucalyptus honey from Mostaganem (M) (42.36 ± 1.14 mg AAE/100 g honey, S27) displayed the highest antioxidant activity, signifying significant effectiveness. The lowest activity was recorded in lavender honey from Sidi Djillali (Tlem) (6.69 ± 0.22 mg AAE/100 g honey, S1).

An interesting observation emerged from the ability to scavenge the ABTS free radical. Both the highest and lowest IC_50_ values were found in mild white mustard honey collected from two distinct geographical locations: Oued Zouzfana (Bechar) (118.12 ± 10.22 mg/mL, S37) and Aïn Fezza (Tlem) (17.97 ± 0.72 mg/mL, S6), as shown in Table S1 and Table 1. Additionally, in the β-carotene bleaching test, the IC_50_ values ranged from 91.80 ± 1.69 mg/mL to 25.23 ± 0.16 mg/mL for euphorbia honey from Ras El Ma (SB) (S22) and mild white mustard honey from Sidi Djillali (Tlem) (S3), respectively (Table 1).

Using Pearson correlation analysis, relationships between the content of bioactive compounds, antioxidant activities, and physicochemical parameters of all NH samples were studied. The results are presented in Table 2. High positive correlations were observed between TPC and TFC (0.734), β-carotene and DPPH (0.766), and β-carotene and ABTS (0.600). In contrast, negative correlations were observed between the bioactive compounds and antioxidant activity, except for the FRAP method, suggesting that phenolic and flavonoid compounds might not be the only factors influencing the bioactivity of honey.

Significant correlations were observed between EC and FRAP (0.532), while negative correlations were recorded between EC and DPPH (−0.415), and EC and β-carotene (−0.392).

**Figure 2:**
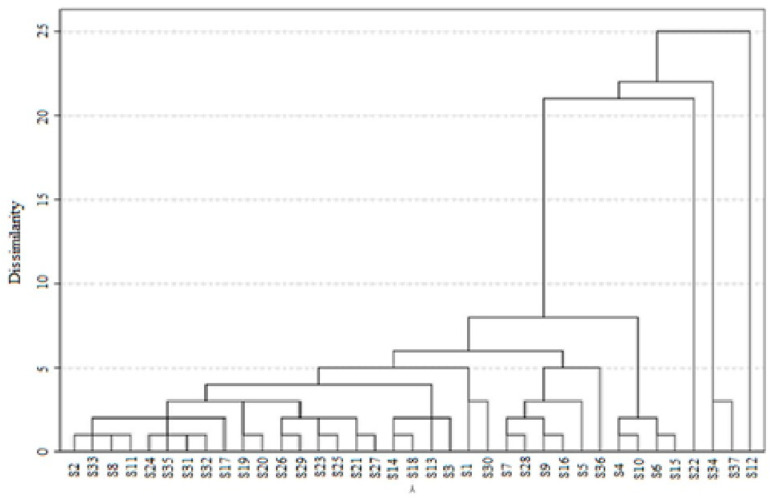
Dendrogram of the cluster analysis using the average Euclidean distance for 37 honey samples collected from the western region of Algeria, in relation to the studied parameters.

To identify NH samples with relatively similar profiles based on the determined parameters (TPC, TFC, pH, EC, DPPH, ABTS, β-carotene, and FRAP values), a cluster analysis was conducted. At a distance of 5, four groups displayed evident homogeneity (Figure 2). The first group consisted of 21 samples (from S2 to S23). By further dividing the first group at a distance of 1, a clear differentiation into 11 sub-groups was achieved. The second group included two samples (S1 and S30). The third group comprised five samples (S7, S28, S9, S16, and S5), which were subdivided into three sub-groups at a distance of 1. The fourth group contained a single sample (S36). These four groups merged into a single cluster at a distance of 8, while the second cluster comprised four samples (S4, S10, S6, and S15). The remaining samples (S22, S34, S37, and S12) formed three separate groups.

### 3.4. Evaluation of Antimicrobial Effects

The antimicrobial activity of the NHs was initially assessed using a well-diffusion assay. Given the number of honey samples and target microorganisms, the inhibitory strength was investigated at high concentrations (80% and 50%) (Table 3). Considerable variation was observed, not only between microorganisms but also among NHs of the same botanical origin.

Regarding microorganisms, active NHs demonstrated stronger activity against Gram-positive bacteria compared to Gram-negative ones. *S. aureus* (MSSA) ATCC 25923 exhibited the highest susceptibility, with DIZ values ranging from 9.00 ± 0.00 to 30.00 ± 0.00 mm. In contrast, the DIZ for *P. aeruginosa* ranged from 8.00 ± 0.00 to 8.67 ± 0.58 mm. No activity was observed against any of the *C. albicans* strains.

Statistical analysis revealed that the concentration of NHs influenced the DIZ in some samples. For instance, the variation in DIZ was highly significant (*p* < 0.001) for multifloral honey (S5) against *S. aureus* (MSSA) ATCC 25923. Additionally, a significant difference (*p* < 0.05) was observed for multifloral (S21) and jujube tree (S32) honeys against the two *S. aureus* strains, whereas no significant variation was detected with *P. aeruginosa* ATCC 27853.

Considering botanical origin, multifloral honey generally displayed potent antibacterial activity, although its effectiveness varied. For example, multifloral honey collected from Sidi Djillali (Tlem) (S5) recorded the highest DIZ of 28.33 ± 0.58 mm, while multifloral honey from Sebaa Chioukh (Tlem) (S14) exhibited the lowest DIZ of 9.00 ± 0.00 mm at an 80% (*w*/*v*) dilution against *S. aureus* (MSSA) ATCC 25923. Similar trends were observed for eucalyptus and mild white mustard honeys.

In a separate assay, gentamicin and amphotericin B were tested against the reference strains. The results, shown in Table 4, confirmed the strains’ sensitivity. Interestingly, the DIZ values of multifloral (S5, S13, and S24) and milk thistle (**∫**) honeys exceeded the upper limit of the CLSI interpretive criteria for *S. aureus* (MSSA) ATCC 25923. These results should be considered indicative rather than conclusive.

**Figure 3:**
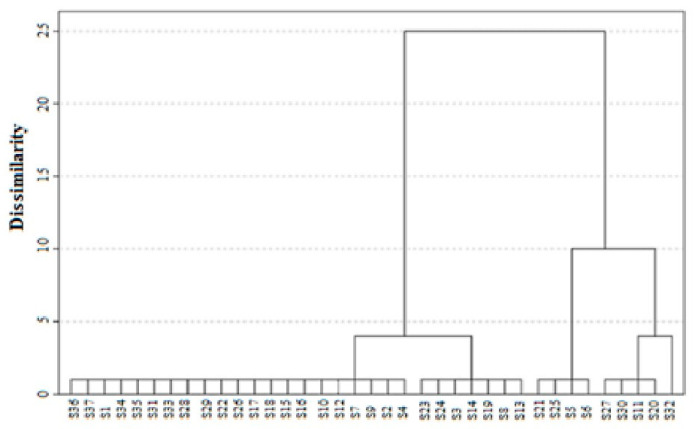
Dendrogram of the effect of honey samples on bacterial strains using the Euclidean distance.

Cluster analysis, based on the interaction between “samples × effect on strains”, classified the NHs into five distinct groups, delimited by a Euclidean distance of 1 (Figure 3). The first group included samples such as S3, S8, S13, S14, S19, S23, and S24, which exhibited antimicrobial activity exclusively against *S. aureus* (MSSA) ATCC 25923. A second group, comprising S5, S6, S21, and S25, demonstrated effectiveness against both *S. aureus* strains. The third group, which included S11, S20, S27, and S30, displayed activity against *S. aureus* (MSSA) ATCC 25923 and *P. aeruginosa* ATCC 27853. Interestingly, the fourth group, represented solely by S32, was the only honey that acted against all three reference bacteria. The remaining samples formed a fifth group that showed no detectable antimicrobial activity. This analysis highlights the variable effectiveness of NHs based on their interactions with bacterial strains.

Further insights were obtained through the determination of MICs and MBCs, as summarized in Table 3. Consistent with the results of the well-diffusion assay, *S. aureus* ATCC 25923 emerged as the most sensitive strain, with MIC values ranging from 60% (*w*/*v*) to 80% (*w*/*v*). Notably, MICs and MBCs were identical in samples such as S3, S6, S11, S14, S19, S20, S21, S24, S27, and S32 or differed by only one dilution step in others like S8, S13, S23, and S30. In contrast, samples S5 and S25 required undiluted honey (100%) to achieve an antibacterial effect. Interestingly, some samples demonstrated equivalent MICs for both *S. aureus* strains (*MSSA* ATCC 25923 and *MRSA* ATCC 43300) or for *S. aureus* (MSSA) ATCC 25923 and *P. aeruginosa* ATCC 27853, as observed with samples S20 and S30.

Although certain samples such as S3, S6, S11, S14, and S27 exhibited relatively low DIZ values in the well-diffusion assay, their MICs were among the lowest recorded, underscoring their potent antibacterial activity. These honeys originated from diverse botanical sources, including multifloral, mild white mustard (*Sinapis alba* L.), thyme (*Thymus vulgaris* L.), and eucalyptus (*Eucalyptus globulus* Labill), reflecting the influence of botanical origin on their bioactive properties.

## 4. Discussion

The west of Algeria is characterized by heterogeneous climatic conditions that have led to a rich biodiversity in endemic flora. Under these conditions, bees produce various types of honey with numerous health benefits. Several studies have reported the antioxidant and antimicrobial properties of Algerian NHs [4,36]. However, previous research has not thoroughly examined the close relationship between these properties and the composition of bioactive compounds.

In the present study, 37 Algerian NHs were collected from diverse regions of western Algeria. The phenolic content of the NHs ranged from 24.17 ± 1.38 to 122.15 ± 3.55 mg GAE/100 g of honey (Table 1). Similar phenolic content levels were previously reported in honeys from Kosovo (49.39 ± 45.88 to 100.95 ± 4.51 mg GAE/100 g) [37] and Tunisia (32.17 ± 0.09 to 119.42 ± 0.25 mg GAE/100 g) [38]. However, these values were lower compared to honeys from Morocco (75.52 ± 7.45 to 245.22 ± 11.40 mg GAE/100 g) [39] and Poland (181.0 ± 5.0 to 355.0 ± 30.0 mg GAE/100 g) [40]. This variation in phenolic content may be influenced by several factors, including the botanical origin of the honey, the year of harvest, and hive environmental conditions [41].

Carob tree honey exhibited the highest phenolic content (122.15 ± 3.55 mg GAE/100 g, S12), whereas lavender honey had the lowest (24.17 ± 1.38 mg GAE/100 g, S1). High phenolic values were also observed in multifloral (82.49 ± 2.06 mg GAE/100 g, S4), mild white mustard (71.18 ± 5.65 mg GAE/100 g, S6), and milk thistle (67.22 ± 4.44 mg GAE/100 g, S23) honeys. Notably, a highly significant difference (*p* < 0.001) was found among all NHs studied. These values were significantly higher than those recorded for acacia and chestnut honeys from Kosovo (25.76 ± 10.16 and 35.77 ± 8.26 mg GAE/100 g, respectively) [37].

The TFC in NH samples varied between 0.07 ± 0.01 and 33.49 ± 4.90 mg QE/100 g of honey (Table 1). The highest flavonoid content was found in carob tree honey (33.49 ± 4.90 mg QE/100 g, S12), followed by multifloral honeys (26.49 ± 3.04 mg QE/100 g, S4; 19.15 ± 2.51 mg QE/100 g, S15) and mild white mustard honey (17.26 ± 2.38 mg QE/100 g, S6). The lowest flavonoid values were observed in lavender (0.07 ± 0.01 mg QE/100 g, S1), thyme (1.63 ± 0.83 mg QE/100 g, S7), and orange tree (1.79 ± 0.27 mg QE/100 g, S28) honeys.

These findings align with previous studies, which reported similar TFC values for honeys from Tunisia (9.58 ± 0.03 to 22.45 ± 0.10 mg QE/100 g) [38], Italy (5.09 ± 2.51 to 14.05 ± 8.03 mg QE/100 g) [42], and Poland (8.00 ± 0.10 to 20.00 ± 0.20 mg QE/100 g) [40]. However, the TFC levels in Algerian NHs were higher than those recorded for Kosovo honeys (1.11 ± 0.62 to 7.51 ± 3.75 mg QE/100 g) [37] and significantly lower than those reported for Moroccan honeys (4.26 ± 1.63 to 139.62 ± 1.63 mg QE/100 g) [43].

Flavonoids represent over 50% of the total natural phenolic compounds and can be transferred to honey, along with other bioactive polyphenolic derivatives, from melliferous plants during nectar collection by bees [44,45]. Additionally, the levels of these compounds are associated with the color variation of honey [46]. Specifically, a significant decrease in flavonoid content was observed, with a reduction from 33.49 ± 4.90 mg QE/100 g (S12) in dark amber honey to 1.79 ± 0.27 mg QE/100 g (S28) in nearly colorless honey. These results demonstrate that Algerian NHs are relatively rich in bioactive phenolic compounds and represent a valuable source of dietary antioxidants.

The antioxidant activity of Algerian NHs was measured using four different assays: DPPH, ABTS, β-carotene, and FRAP. Overall, the data showed that NHs exhibited antioxidant activity that varied widely (Table 1).

The DPPH radical scavenging activity values varied significantly among the NHs studied, ranging from 17.73 ± 1.53 to 98.58 ± 6.29 mg/mL (Table 1). The highest activity (i.e., lower IC_50_ values) was observed for multifloral (17.73 ± 1.53, S13; 22.91 ± 0.91 mg/mL, S15) and mild white mustard (22.44 ± 0.54 mg/mL, S6) honeys, while euphorbia honey (98.58 ± 6.29 mg/mL, S22) exhibited the lowest activity.

The results of the current study are consistent with those reported by Boussaid et al. [38] for Tunisian honeys from orange, thyme, eucalyptus, rosemary, horehound, and mint plants (ranging from 11.08 ± 0.32 to 93.26 ± 0.37 mg/mL) and by Gül et al. [47] for Turkish monofloral honeys (ranging from 12.01 ± 0.35 to 65.52 ± 0.88 mg/mL). However, these values were lower than those reported for Portuguese monofloral honeys (ranging from 84.98 ± 1.19 to 168.94 ± 19.20 mg/mL) [46].

Additionally, stronger correlations were found between DPPH radical scavenging activity and both TPC and TFC. In this study, mild white mustard honey (S6), which contains high concentrations of TPC (71.18 ± 5.65 mg GAE/100 g) and TFC (17.26 ± 2.38 mg QE/100 g), showed the lowest IC_50_ value, indicating the highest antioxidant activity. Furthermore, it was observed that DPPH values and total phenolic compounds were lower in lighter-colored honeys, such as rosemary (S2 and S29), thyme (S7), and orange tree (S16 and S28), compared with darker-colored honeys like carob tree (S12) and multifloral (S13, S19, and S20), which also exhibited high mean values for electrical conductivity (EC > 0.70 mS/cm) (Table 1). Overall, our results align perfectly with those reported in several other studies [38,46,47].

The results for the ABTS assay ranged from 17.97 ± 0.72 to 118.12 ± 10.22 mg/mL (Table 1). The highest radical scavenging activity was observed in mild white mustard and sage honeys (118.12 ± 10.22, S37; 100.62 ± 9.13 mg/mL, S34, respectively), while the lowest values were found in carob tree (24.17 ± 0.80, S10; 27.12 ± 0.70 mg/mL, S12) and multifloral (24.51 ± 0.57 mg/mL, S15) honeys. The data obtained were similar to those reported by Silva et al. [32] for Brazilian honeys (ranging from 21.2 ± 0.3 to 53.1 ± 0.8 mg/mL) but were higher than those reported for Palestinian honeys (ranging from 3.26 ± 0.20 to 16.28 ± 0.25 mg/mL) [48] and Moroccan honeys (ranging from 4.63 ± 0.62 to 31.00 ± 0.62 mg/mL) [43].

In general, honeys that exhibited higher ABTS+ scavenging activity were also more effective in the DPPH assay, as confirmed by the correlation between these two variables.

Antioxidant activity measured by the β-carotene bleaching test ranged between 25.23 ± 0.16 and 91.80 ± 1.69 mg/mL, a result similar to that found in the DPPH assay. The lowest value, indicating the highest antioxidant activity, was observed in multifloral honey samples (25.23 ± 0.16, S3; 27.24 ± 0.55, S20; 29.20 ± 0.13 mg/mL, S15), while the highest values were found in euphorbia (91.80 ± 1.69 mg/mL, S22) and mild white mustard (85.22 ± 0.68 mg/mL, S37) honeys. Our data were similar to those reported by Ferreira et al. [46] for monofloral honeys obtained in Northeast Portugal (ranging from 12.01 ± 0.96 to 75.51 ± 0.04 mg/mL) and by Bouhlali et al. [15] for Moroccan monofloral honeys (ranging from 24.37 ± 0.28 to 79.08 ± 0.62 mg/mL).

Antioxidant activity determined by the FRAP assay ranged from 6.69 ± 0.22 to 42.36 ± 1.14 mg AAE/100 g, depending on the honey type (Table 1).

The observed differences between honeys can be partly attributed to variations in the relative quantities of minor bioactive compounds, including phenolic acids, flavonoids, enzymes (e.g., glucose oxidase, catalase), vitamins (e.g., ascorbic acid), and trace elements. Although these compounds are present in small quantities, they play a critical role in the antioxidant effect.

According to Mayer et al. [49], antioxidant activity primarily depends on the floral source of honey. However, it is not the sole factor contributing to its antioxidant properties. In fact, differences in antioxidant activity can be attributed to the presence of various polyphenolic compounds, such as flavonoids, phenolic acids, and other phenolic compounds, each contributing distinct antioxidant effects. Typically, darker honey samples exhibit higher antioxidant activity, with variations attributed to the quantitative and qualitative nature of their polyphenolic content [50]. Furthermore, Taormina et al. [51] reported a correlation between phenolic antioxidants and the antibacterial activity of honey. The findings of this study raise the question of whether the antimicrobial activity of Algerian NHs is linked to their bioactive compound content.

The well-diffusion assay was employed to evaluate the antimicrobial properties of 37 Algerian NHs. This simple and cost-effective method effectively differentiates honeys with antimicrobial activity from those without. The findings indicated that Algerian NHs possess notable antibacterial activity, with *S. aureus* (MSSA) ATCC 25923 identified as the most sensitive strain. This aligns with previous studies reporting greater sensitivity of Gram-positive bacteria compared to Gram-negative ones, likely due to differences in cell wall composition [1]. The thick peptidoglycan layer in Gram-positive bacteria facilitates the penetration of active agents.

According to Küçük et al.’s classification scale [34], the antibacterial activity of NHs varies by concentration. At 80% (*w*/*v*), NHs were classified as high or moderate antibacterial agents, whereas at 50% (*w*/*v*), the same samples were categorized as moderate or low agents. However, at 80% (*w*/*v*), NHs exhibited very low activity against *P. aeruginosa* ATCC 27853.

Algerian NHs demonstrated dose-dependent antibacterial activity, with higher concentrations (e.g., 80% *w*/*v*) being more effective. These results are consistent with findings by Ghramh et al. [33], who showed that Saudi honeys inhibited Gram-negative bacteria at 40−80% concentrations. Similarly, Laallam et al. [4] observed that Algerian NHs from the Sahara displayed antimicrobial activity in their natural state without dilution.

Botanical origin significantly influences the antibacterial potency of Algerian NHs. Variations in antibacterial activity were observed even among honeys derived from the same botanical source, suggesting the influence of other factors. Low pH and EC, directly tied to botanical sources, enhance antimicrobial effects synergistically. Low pH disrupts bacterial cell membranes, denatures proteins, and interferes with enzymes regulating pH homeostasis, thereby impairing bacterial survival [52,53]. Meanwhile, low EC reflects reduced ion concentration, destabilizing osmotic balance and bacterial membranes [52,53,54,55]. The study confirmed that stronger antibacterial effects were associated with NHs exhibiting lower EC and pH values.

Phenolic compounds and flavonoids also play a critical role in antimicrobial activity. These bioactive compounds inhibit bacterial growth via multiple mechanisms. High concentrations denature proteins, while low concentrations interfere with bacterial energy production by inhibiting enzymes, altering membrane permeability [54,56,57]. In this study, NH samples with TPC > 32.87 ± 2.83 mg GAE/100 g and TFC > 5.53 ± 0.76 mg QE/100 g showed strong antibacterial effects against at least two bacterial strains, regardless of Gram classification.

The antibacterial activity of NHs is multifactorial, influenced by bioactive compounds, physicochemical properties, and other factors. Hydrogen peroxide (H_2_O_2_) and bee defensin-1 likely contribute to this activity but were not investigated in this study, highlighting the need for further research [1,2].

MIC and MBC assays were conducted to ensure accurate results regarding antibacterial activity. A weak correlation between well-diffusion and MIC results was noted, particularly in samples S3, S6, S11, S14, and S27. Similar findings by Osés et al. [58] and Hussain et al. [59] attribute these discrepancies to limited diffusion of high-molecular-weight or non-polar compounds in agar. These limitations underline the importance of complementing the well-diffusion method with MIC for a comprehensive assessment.

Interestingly, some NHs exhibited identical MIC values against both MSSA and MRSA, as well as between Gram-positive and Gram-negative bacteria, suggesting broad-spectrum activity. Active NHs primarily demonstrated bactericidal effects. Their notable efficacy against MRSA and *P. aeruginosa* highlights the therapeutic potential of west Algerian NHs in combating antibiotic-resistant pathogens. Remarkably, no bacterial resistance to honey has been reported, attributed to its multifactorial composition and diverse mechanisms targeting multiple bacterial sites [16].

Lastly, combining antioxidant and antibacterial analyses identified sample S6 (*Sinapis alba* L.) as the most beneficial NH. This honey exhibited strong antioxidant properties (TPC: 71.18 ± 5.65 mg GAE/100 g; TFC: 17.26 ± 2.38 mg QE/100 g) and antimicrobial activity. Its favorable physicochemical properties (pH = 3.99 ± 0.05; EC = 0.553 ± 0.001 mS/cm) further distinguish it. These findings emphasize the close relationship between bioactive compounds and antibacterial activity, contributing to a deeper understanding of the bioactive properties of Algerian honey.

## 5. Conclusions

To summarize, west Algerian honeys demonstrated substantial antioxidant and antibacterial activities. This study highlighted variability in TPC and TFC contents across samples, which were strongly correlated with antibacterial potency. Interestingly, inverse correlations were identified between bioactive compound levels and antioxidant activity, consistent with the initial objectives of the study. The observed variability in composition and biological activity was primarily attributed to the botanical origin of the honey samples. Among these, sample S6, derived from mild white mustard, exhibited remarkable bioactivity, positioning it as a promising candidate for therapeutic applications. Its potent antibacterial activity underscores its potential for addressing bacterial infections, particularly those involving antibiotic-resistant strains, as well as for wound care management. Future investigations should prioritize detailed profiling of phenolic compounds using HPLC-MS, explore the contributions of peroxidic and non-peroxidic factors (e.g., bee defensin-1), and conduct in vivo studies to substantiate its therapeutic potential. Furthermore, this study reaffirmed that the broth dilution method remains the most reliable approach for accurately assessing the antibacterial activity of honey, offering a robust foundation for future research into therapeutic honeys.

## Figures and Tables

**Table 1:** TPC, TFC, pH, EC, DPPH, ABTS, β-Carotene, and FRAP values of the analyzed honey samples.

N°	TPC * (mg GAE/100 g)	TFC * (mg QE/100 g)	pH *	EC * (mS/cm)	DPPH Scavenging Activity * IC_50_ (mg/mL)	ABTS Assay * IC_50_ (mg/mL)	β-Carotene * IC_50_ (mg/mL)	FRAP Value * (mg AAE/100 g Honey)
S1	24.17 ± 1.38	0.07 ± 0.01	3.61 ± 0.03	0.445 ± 0.006	33.05 ± 0.61	64.86 ± 2.94	56.50 ± 0.96	6.69 ± 0.22
S2	39.14 ± 2.81	6.11 ± 0.72	3.38 ± 0.06	0.259 ± 0.001	26.05 ± 0.79	33.47 ± 0.49	38.70 ± 0.48	14.07 ± 0.44
S3	57.64 ± 1.20	2.47 ± 0.45	3.88 ± 0.03	0.209 ± 0.001	28.09 ± 1.67	39.86 ± 1.49	25.23 ± 0.16	16.08 ± 0.17
S4	82.49 ± 2.06	26.49 ± 3.04	4.96 ± 0.02	0.276 ± 0.005	32.85 ± 1.54	31.93 ± 2.58	34.67 ± 0.20	18.49 ± 0.37
S5	42.91 ± 0.71	9.35 ± 1.65	5.62 ± 0.04	0.250 ± 0.002	61.60 ± 1.23	71.58 ± 7.79	69.10 ± 6.28	16.72 ± 0.53
S6	71.18 ± 5.65	17.26 ± 2.38	3.99 ± 0.05	0.553 ± 0.001	22.44 ± 0.54	17.97 ± 0.72	36.37 ± 0.40	15.94 ± 1.06
S7	57.45 ± 3.18	1.63 ± 0.83	4.30 ± 0.01	0.293 ± 0.001	49.17 ± 4.42	49.54 ± 2.03	56.47 ± 1.26	26.06 ± 0.70
S8	54.82 ± 4.43	4.85 ± 0.57	4.28 ± 0.06	0.470 ± 0.006	29.22 ± 2.06	34.59 ± 0.61	42.91 ± 0.20	17.99 ± 0.92
S9	40.19 ± 5.47	9.08 ± 0.75	4.64 ± 0.01	0.254 ± 0.028	60.39 ± 1.96	48.27 ± 1.25	68.86 ± 0.68	18.00 ± 0.62
S10	77.77 ± 3.64	19.10 ± 1.41	4.50 ± 0.01	0.415 ± 0.018	34.66 ± 0.38	24.17 ± 0.80	37.50 ± 0.31	29.01 ± 1.18
S11	44.25 ± 4.02	5.76 ± 0.80	4.60 ± 0.01	0.445 ± 0.008	35.23 ± 1.24	36.14 ± 0.20	47.65 ± 0.16	18.05 ± 0.44
S12	122.15 ± 3.55	33.49 ± 4.90	4.36 ± 0.01	0.725 ± 0.013	27.25 ± 0.81	27.12 ± 0.70	31.92 ± 0.41	34.40 ± 0.61
S13	52.23 ± 0.98	13.37 ± 2.27	4.11 ± 0.02	0.910 ± 0.008	17.73 ± 1.53	57.39 ± 2.52	32.38 ± 0.16	39.79 ± 0.86
S14	56.55 ± 2.62	9.54 ± 1.21	4.63 ± 0.05	0.518 ± 0.008	27.14 ± 0.44	37.82 ± 0.40	34.04 ± 0.14	34.59 ± 1.26
S15	57.23 ± 2.75	19.15 ± 2.51	4.28 ± 0.01	0.380 ± 0.005	22.91 ± 0.91	24.51 ± 0.57	29.20 ± 0.13	13.78 ± 0.27
S16	38.25 ± 1.64	2.40 ± 0.35	4.28 ± 0.02	0.165 ± 0.017	59.29 ± 3.52	35.92 ± 0.81	55.07 ± 0.79	14.53 ± 0.18
S17	48.90 ± 2.95	2.22 ± 0.50	4.62 ± 0.05	0.421 ± 0.002	44.14 ± 0.76	48.90 ± 1.22	38.17 ± 0.29	14.47 ± 0.26
S18	67.22 ± 4.44	4.79 ± 0.73	3.77 ± 0.10	0.321 ± 0.002	29.11 ± 2.37	49.20 ± 1.69	32.84 ± 0.13	34.65 ± 0.50
S19	39.62 ± 1.06	5.04 ± 0.11	5.12 ± 0.08	1.196 ± 0.021	25.94 ± 0.41	37.75 ± 0.57	37.66 ± 0.12	31.90 ± 2.32
S20	32.87 ± 2.83	5.53 ± 0.76	4.40 ± 0.00	1.040 ± 0.002	37.93 ± 2.69	28.40 ± 0.12	27.24 ± 0.55	35.06 ± 0.65
S21	61.57 ± 3.48	7.11 ± 0.94	4.19 ± 0.01	0.498 ± 0.001	38.58 ± 0.86	33.28 ± 0.79	44.88 ± 0.70	39.27 ± 0.75
S22	55.04 ± 1.49	8.57 ± 1.33	4.51 ± 0.65	0.236 ± 0.005	98.58 ± 6.29	39.15 ± 0.61	91.80 ± 1.69	14.57 ± 0.58
S23	48.69 ± 1.86	13.37 ± 0.78	4.27 ± 0.01	0.436 ± 0.006	50.36 ± 1.01	36.75 ± 1.22	50.43 ± 0.31	26.32 ± 1.04
S24	33.50 ± 2.29	4.40 ± 1.22	3.88 ± 0.01	0.163 ± 0.002	40.47 ± 0.13	33.30 ± 0.82	40.06 ± 0.06	17.36 ± 1.13
S25	43.34 ± 2.31	9.40 ± 1.25	4.10 ± 0.01	0.320 ± 0.002	49.36 ± 0.68	41.21 ± 0.68	42.43 ± 0.25	29.23 ± 0.99
S26	28.37 ± 0.60	6.57 ± 0.39	4.70 ± 0.03	0.317 ± 0.003	40.76 ± 1.78	45.94 ± 0.83	45.77 ± 0.66	40.74 ± 0.48
S27	46.53 ± 2.73	10.84 ± 1.55	4.29 ± 0.04	0.575 ± 0.010	43.62 ± 1.25	32.29 ± 0.24	47.44 ± 0.77	42.36 ± 1.14
S28	41.95 ± 0.96	1.79 ± 0.27	4.65 ± 0.02	0.210 ± 0.001	47.34 ± 1.26	58.69 ± 0.77	59.76 ± 1.07	18.81 ± 0.79
S29	36.52 ± 1.12	2.94 ± 0.61	4.19 ± 0.02	0.249 ± 0.001	47.45 ± 0.63	49.37 ± 0.42	49.28 ± 0.79	36.89 ± 0.61
S30	36.37 ± 1.10	13.37 ± 1.90	4.00 ± 0.02	0.363 ± 0.001	25.88 ± 1.80	52.60 ± 2.58	41.58 ± 0.48	14.30 ± 0.20
S31	48.47 ± 3.13	13.34 ± 1.91	3.94 ± 0.01	0.447 ± 0.004	45.13 ± 0.62	24.02 ± 0.50	46.41 ± 0.56	16.58 ± 0.41
S32	40.27 ± 2.58	12.65 ± 1.80	4.69 ± 0.02	0.248 ± 0.018	54.80 ± 0.77	34.28 ± 0.38	43.53 ± 3.78	15.28 ± 0.53
S33	42.22 ± 1.73	7.33 ± 0.91	5.31 ± 0.01	0.226 ± 0.002	33.75 ± 2.59	30.07 ± 0.22	36.96 ± 4.15	11.08 ± 0.84
S34	42.09 ± 1.81	11.27 ± 1.21	4.56 ± 0.05	0.202 ± 0.001	49.27 ± 0.23	100.62 ± 9.13	69.34 ± 0.80	13.10 ± 0.42
S35	36.43 ± 1.16	9.64 ± 0.66	4.99 ± 0.01	0.236 ± 0.001	46.51 ± 2.20	29.23 ± 0.29	33.64 ± 0.54	11.02 ± 0.58
S36	32.23 ± 1.66	3.35 ± 0.91	5.01 ± 0.06	0.464 ± 0.006	67.49 ± 0.68	73.33 ± 1.71	41.11 ± 1.03	22.44 ± 0.88
S37	49.85 ± 3.12	4.01 ± 0.42	4.44 ± 0.01	0.249 ± 0.001	58.53 ± 1.64	118.12 ± 10.22	85.22 ± 0.65	12.45 ± 0.35

Each value is the mean ± SD (n = 3). TPC: total phenolic content determined by the Folin–Ciocalteu method; TFC: total flavonoids content determined by AlCl_3_ coloration; pH; EC: electrical conductivity; DPPH; ABTS; β-Carotene bleaching inhibition; FRAP. IC_50_: concentration causing 50% inhibition. *: indicates a high significant difference (*p* < 0.001).

**Table 2:** Correlation matrix of the studied parameters (Pearson correlation coefficients).

	TPC	TFC	pH	EC	DPPH	ABTS	β-Carotene	FRAP Value
**TPC**	1							
**TFC**	0.734 **	1						
**pH**	−0.046	0.093	1					
**EC**	0.152	0.179	0.034	1				
**DPPH**	−0.269	−0.260	0.328 *	−0.415 *	1			
**ABTS**	−0.286	−0.344 *	0.141	−0.214	0.330 *	1		
**β-Carotene**	−0.229	−0.262	0.165	−0.392 *	0.766 **	0.600 **	1	
**FRAP value**	0.229	0.163	−0.062	0.532 **	−0.218	−0.207	−0.321	1

Pearson correlation between total phenolic content (TPC), total flavonoids content (TFC), pH, electrical conductivity (EC) and antioxidant activities with DPPH, ABTS, β-Carotene bleaching inhibition, and ferric reducing/antioxidant power (FRAP). *: correlation is significant at the 0.05 level (2-tailed), **: correlation is significant at the 0.01 level (2-tailed).

**Table 3:** Antimicrobial activity in honeys in the western region of Algeria.

	Gram-Positive										Gram-Negative				
	*Staphylococcus aureus* ATCC 43300					*Staphylococcus aureus* ATCC 25923					*Pseudomonas aeruginosa* ATCC 27853				
	DIZ ^a^		*Sig.* ^b^	MIC ^c^	MBC ^d^	DIZ		*Sig.*	MIC	MBC	DIZ		*Sig.*	**MIC**	**MBC**
N°	80% (*w*/*v*) ^e^	50% (*w*/*v*)		[%]	[%]	80% (*w*/*v*)	50% (*w*/*v*)		[%]	[%]	80% (*w*/*v*)	50% (*w*/*v*)		**[%]**	**[%]**
S1	/	/	-	/	/	/	/	-	/	/	/	/	-	/	/
S2	/	/	-	/	/	/	/	-	/	/	/	/	-	/	/
S3	/	/	-	/	/	12.33 ± 0.58	9.33 ± 0.58	*	60.00 ± 0.00	60.00 ± 0.00	/	/	-	/	/
S4	/	/	-	/	/	/	/	-	/	/	/	/	-	/	/
S5	13.33 ± 1.15	10.00 ± 2.00	NS	80.00 ± 0.00	80.00 ± 0.00	28.33 ± 0.58	25.00 ± 0.00	**	100.00 ± 0.00	100.00 ± 0.00	/	/	-	/	/
S6	14.33 ± 0.58	11.67 ± 0.58	*	60.00 ± 0.00	60.00 ± 0.00	14.00 ± 1.73	11.33 ± 1.15	NS	60.00 ± 0.00	60.00 ± 0.00	/	/	-	/	/
S7	/	/	-	/	/	/	/	-	/	/	/	/	-	/	/
S8	/	/	-	/	/	13.33 ± 0.58	11.67 ± 1.15	NS	80.00 ± 0.00	100.00 ± 0.00	/	/	-	/	/
S9	/	/	-	/	/	/	/	-	/	/	/	/	-	/	/
S10	/	/	-	/	/	/	/	-	/	/	/	/	-	/	/
S11	/	/	-	/	/	10.33 ± 1.53	8.67 ± 1.53	NS	80.00 ± 0.00	80.00 ± 0.00	8.00 ± 0.00	/	-	60.00 ± 0.00	60.00 ± 0.00
S12	/	/	-	/	/	/	/	-	/	/	/	/	-	/	/
S13	/	/	-	/	/	15.67 ± 0.58	13.33 ± 1.15	*	80.00 ± 0.00	100.00 ± 0.00	/	/	-	/	/
S14	/	/	-	/	/	9.00 ± 0.00	/	-	60.00 ± 0.00	60.00 ± 0.00	/	/	-	/	/
S15	/	/	-	/	/	/	/	-	/	/	/	/	-	/	/
S16	/	/	-	/	/	/	/	-	/	/	/	/	-	/	/
S17	/	/	-	/	/	/	/	-	/	/	/	/	-	/	/
S18	/	/	-	/	/	/	/	-	/	/	/	/	-	/	/
S19	/	/	-	/	/	12.33 ± 0.58	9.33 ± 0.58	*	80.00 ± 0.00	80.00 ± 0.00	/	/	-	/	/
S20	/	/	-	/	/	13.00 ± 1.00	11.33 ± 0.58	NS	80.00 ± 0.00	80.00 ± 0.00	8.00 ± 0.00	/	-	80.00 ± 0.00	80.00 ± 0.00
S21	14.33 ± 0.57	8.33 ± 1.53	*	80.00 ± 0.00	80.00 ± 0.00	14.33 ± 0.58	12.67 ± 0.58	*	80.00 ± 0.00	80.00 ± 0.00	/	/	-	/	/
S22	/	/	-	/	/	/	/	-	/	/	/	/	-	/	/
S23	/	/	-	/	/	30.00 ± 0.00	25.00 ± 0.00	-	80.00 ± 0.00	100.00 ± 0.00	/	/	-	/	/
S24	/	/	-	/	/	17.67 ± 1.15	14.67 ± 1.15	*	80.00 ± 0.00	80.00 ± 0.00	/	/	-	/	/
S25	14.33 ± 0.57	10.00 ± 1.00	*	100.00 ± 0.00	100.00 ± 0.00	12.33 ± 0.58	10.00 ± 1.73	NS	80.00 ± 0.00	80.00 ± 0.00	/	/	-	/	/
S26	/	/	-	/	/	/	/	-	/	/	/	/	-	/	/
S27	/	/	-	/	/	10.33 ± 1.53	8.33 ± 1.53	NS	60.00 ± 0.00	60.00 ± 0.00	8.00 ± 0.00	/	-	80.00 ± 0.00	80.00 ± 0.00
S28	/	/	-	/	/	/	/	-	/	/	/	/	-	/	/
S29	/	/	-	/	/	/	/	-	/	/	/	/	-	/	/
S30	/	/	-	/	/	13.33 ± 1.53	10.33 ± 1.53	NS	80.00 ± 0.00	100.00 ± 0.00	8.67 ± 0.58	/	-	80.00 ± 0.00	80.00 ± 0.00
S31	/	/	-	/	/	/	/	-	/	/	/	/	-	/	/
S32	11.33 ± 1.53	8.67 ± 1.53	*	60.00 ± 0.00	60.00 ± 0.00	12.33 ± 0.58	9.33 ± 0.58	*	100.00 ± 0.00	100.00 ± 0.00	8.33 ± 0.58	/	-	60.00 ± 0.00	60.00 ± 0.00
S33	/	/	-	/	/	/	/	-	/	/	/	/	-	/	/
S34	/	/	-	/	/	/	/	-	/	/	/	/	-	/	/
S35	/	/	-	/	/	/	/	-	/	/	/	/	-	/	/
S36	/	/	-	/	/	/	/	-	/	/	/	/	-	/	/
S37	/	/	-	/	/	/	/	-	/	/	/	/	-	/	/

Values were expressed as mean ± SD (n = 3); ATCC: American Type Culture Collection. ^a^ DIZ: diameter of inhibition zone in mm including the disc diameter (6 mm). ^b^ Sig.: significance codes. ^c^ MIC: Minimum Inhibitory Concentration expressed in %. ^d^ MBC: Minimum Bactericidal Concentration expressed in %. ^e^: concentrations of honey samples S(1–37). *: indicates a significant difference (*p* < 0.05); **: indicates a high significant difference (*p* < 0.001); NS: not significant (*p* > 0.05); -: indicates absence of variance between experimental repeats; */*: no antimicrobial activity.

**Table 4:** Antimicrobial activities of antibiotics against microorganisms studied.

Microorganisms	Antibiotics ^a^	
	DIZ ^b^	
	Gent ^a^ (10 µg)	AMB ^a^ (100 µg)
Gram-positive		
*Staphylococcus aureus* ATCC43300	25.00 ± 0.00	/
*Staphylococcus aureus* ATCC25923	27.00 ± 0.00	/
Gram-negative		
*Pseudomonas aeruginosa* ATCC 27853	21.00 ± 0.00	/
Yeast		
*Candida albicans* ATCC10231	/	27.50 ± 3.54
*Candida albicans* ATCC26790	/	31.00 ± 0.33
*Candida albicans* IPP444	/	26.00 ± 0.33

Values were expressed as mean ± SD (n = 3); ATCC: American Type Culture Collection. ^a^ Antibiotics: Gent: Gentamicin; AMB: Amphotericin B; ^b^ DIZ: diameter of inhibition zone in mm, /: No antimicrobial activity.

## Data Availability

The original contributions presented in this study are included in the article/Supplementary Material. Further inquiries can be directed to the corresponding authors.

## Supplementary Materials

The following supporting information can be downloaded at: https://www.mdpi.com/article/10.3390/foods13244120/s1, Table S1. Data of natural honey samples: botanic origin, geographical situation, climate, and harvest season.
