# Supplementary material for: Bioactive Compounds, Antioxidant Properties, and Antimicrobial Profiling of a Range of West Algerian Honeys: In Vitro Comparative Screening Prior to Therapeutic Purpose
Source: Foods. 2024 Dec 20;13(24):4120. doi: 10.3390/foods13244120 (PMC11675739; doi:10.3390/foods13244120)
Supplement: Supplementary file 1 [file foods-13-04120-s001.zip › foods-3340937-supplementary.pdf]

Supplementary Data_Table S1. Data of natural honey samples: botanic origin, geographical situation, climate, and harvest season.

N°	Honey types	Location	GPS coordinates	Climate	Month and year
S1	Lavender (*Lavandula vera* D.C.)	Sidi Djillali (Tlem)	34° 28' 00'' N 1° 34' 60'' O	Subhumid	Beginning of summer
S2	Rosemary (*Rosmarinus officinalis* L.)	Sidi Djillali (Tlem)	34° 28' 00'' N 1° 34' 60'' O		End of spring
S3	Multifloral	Sidi Djillali (Tlem)	34° 28' 00'' N 1° 34' 60'' O		End of spring
S4	Multifloral	Sidi Djillali (Tlem)	34° 28' 00'' N 1° 35' 00'' O		Beginning of summer
S5	Multifloral	El Aricha (Tlem)	34° 13' 22'' N 1° 15' 21'' O		Beginning of summer
S6	Mild white mustard (*Sinapis alba* L.)	Aïn Fezza (Tlem)	34° 52' 45'' N 1° 14' 18'' O		Beginning of summer
S7	Thyme (*Thymus vulgaris* L.)	Beni Snous (Tlem)	34° 38' 35'' N 1° 33' 41'' O		End of spring
S8	Milk thistle (*Silybum marianum* (L.) Gaertn.)	Beni Snous (Tlem)	34° 38' 35'' N 1° 33' 41'' O		Beginning of summer
S9	Multifloral	Oued Chouly (Tlem)	34° 56' 52'' N 1° 03' 17'' O		End of autumn
S10	Carob tree (*Ceratonia siliqua* L.)	Oued Chouly (Tlem)	34° 56' 52'' N 1° 03' 17'' N		End of autumn
S11	Thyme (*Thymus vulgaris* L.)	Beni Mester (Tlem)	34° 52' 00'' N 1° 25' 00'' O		End of spring
S12	Carob tree (*Ceratonia siliqua* L.)	Béni Ghazli (Tlem)	34° 52' 34'' N 1° 07' 56'' O		End of spring
S13	Multifloral	Oued es Safsâf (Tlem)	34° 55' 60'' N 1° 18' 00'' O		Beginning of summer
S14	Multifloral	Sebaa Chioukh (Tlem)	35° 09' 50'' N 1° 21' 27'' O		End of spring
S15	Multifloral	Hennaya (Tlem)	34° 57' 00'' N 1° 22' 00'' O		Beginning of summer
S16	Orange tree (*Citrus sinensis* L.)	Remchi (Tlem)	35° 03' 00'' N 1° 26' 00'' O		End of spring
S17	Multifloral	Honaïne (Tlem)	35° 10' 35'' N 1° 39' 18'' O		End of spring
S18	Milk thistle (*Silybum marianum* (L.) Gaertn.)	Honaïne (Tlem)	35° 10' 35'' N 1° 39' 18'' O		Beginning of summer
S19	Multifloral	Oulhaça El Gherarba (AT)	35° 13' 00'' N 1° 31' 00'' O	Subhumid	End of spring
S20	Multifloral	Beni Ghanem (AT)	35° 15' 16'' N 1° 25' 38'' O		Beginning of summer
S21	Multifloral	Bouzedjar (AT)	35° 34' 28'' N 1° 10' 01'' O		End of spring
S22	Euphorbia (*Euphorbia* L.)	Ras El Ma (SB)	34° 29' 51'' N 0° 49' 10'' O	Semiarid	End of spring
S23	Milk thistle (*Silybum marianum* (L.) Gaertn.)	Telagh (SB)	34° 47' 06'' N 0° 32' 40'' O		End of spring
S24	Multifloral	Lamtâr (SB)	35° 04' 14'' N 0° 47' 53'' O		End of spring
S25	Eucalyptus (*Eucalyptus globulus* Labill.)	Sidi Brahim (SB)	35° 15' 38'' N 0° 34' 03'' O		End of spring
S26	Camphor (*Cinnamomum camphora* L.)	Sidi Ali (M)	36° 06' 17'' N 0° 25' 24'' E	Semiarid	End of autumn

S27	Eucalyptus (*Eucalyptus globulus* Labill.)	Mostaganem (M)	35° 56' 00" N 0° 05' 00" E		Beginning of summer
S28	Orange tree (*Citrus sinensis* L.)	Bouguirat (M)	35° 45' 05" N 0° 15' 12" E		End of spring
S29	Rosemary (*Rosmarinus officinalis* L.)	Djebel Stamboul (Mas)	35° 23' 00" N 0° 09' 00" E	Semiarid	End of spring
S30	Multifloral	Tiaret (Tiaret)	34° 55' 00" N 1° 34' 60" E	Semiarid	End of spring
S31	Multifloral	Aïn Sefra (Naâma)	32° 45' 20" N 0° 35' 09" O	Arid	End of spring
S32	Jujube tree (*Ziziphus lotus* L.)	Aïn Sefra (Naâma)	32° 45' 20" N 0° 35' 09" O		End of spring
S33	Jujube tree (*Ziziphus lotus* L.)	Aïn Ben Khelil (Naâma)	33° 17' 25" N 0° 45' 51" O		End of spring
S34	Sage (*Salvia officinalis* L.)	Naâma (Naâma)	33° 17' 25" N 0° 45' 51" O		End of spring
S35	Harmal (*Peganum harmala* L.)	Mecheria (Naâma)	33° 33' 00" N 0° 17' 00" O		End of spring
S36	Multifloral	Djebel Antar (Bechar)	31° 56' 34" N 1° 55' 52" O	Arid	Beginning of winter
S37	Mild white mustard (*Sinapis alba* L.)	Oued Zouzfana (Bechar)	32° 04 '01" N 1° 14' 27" O		End of spring

S: Sample; **Tlem**: Tlemcen, S(1-18); **AT**: Ain-Temouchent, S(19-21); **SB**: Sidi Bel Abbes, S(22-25); **M**: Mostaganem, S(26-28); **Mas**: Mascara, S(29); Tiaret, S(30); Naâma, S(31-35); Bechar, S(36-37).
